# Association Between Loneliness, eHealth Literacy, and Quality of Life Among Chinese Older Adults: Cross-Sectional Study

**DOI:** 10.2196/88600

**Published:** 2026-05-11

**Authors:** Shouchuang Zhang, Jiayi Li, Jia Tang, Weiyan Jian, Qing Liu, Jing Guo

**Affiliations:** 1 Department of Health Policy and Management, School of Public Health, Peking University, 38 Xue Yuan Rd, Haidian District, Beijing, Beijing, 100191, China, +86 18086471505.; 2 School of Population and Health, Renmin University of China, Beijing, Beijing, China; 3 Bigdata and Responsible Artificial Intelligence for National Governance, Renmin University of China, Beijing, Beijing, China; 4 Key Laboratory of Health System Reform and Governance, National Health Commission of China, Beijing, Beijing, China; 5 General Practice Department, Second Outpatient Section, Peking University Third Hospital, Beijing, Beijing, China

**Keywords:** loneliness, quality of life, eHealth literacy, health of older adults, China, cross-sectional study

## Abstract

**Background:**

Loneliness is a growing public health challenge among older adults and is associated with a wide range of adverse health outcomes. The role of eHealth literacy in shaping the relationship between loneliness and quality of life remains unclear.

**Objective:**

This study aimed to examine the contributing role of eHealth literacy in moderating the associations between loneliness and quality of life among older adults.

**Methods:**

A community-based survey was conducted in 2025 among older adults aged 60 years or older living in northwestern China. A total of 2110 participants were included. Multiple linear regression and interaction models were used to assess associations and moderating effects. Sensitivity analyses were conducted by replacing the outcome variable with depressive symptoms and by performing stratified analyses to assess the robustness of the primary interaction findings.

**Results:**

Loneliness showed a consistent negative association with quality of life (β=−0.83, 95% CI −1.18 to −0.49; *P*<.001). Higher overall eHealth literacy was associated with better quality of life (β=0.41, 95% CI 0.35-0.47; *P*<.001). Interaction models indicated that higher eHealth literacy was associated with a steeper negative association between loneliness and quality of life (β=−0.04, 95% CI −0.08 to −0.002; *P*=.04). Sensitivity analyses produced similar results across alternative outcomes and subgroups.

**Conclusions:**

Higher loneliness was related to a poorer quality of life. Higher eHealth literacy was associated with a steeper negative association between loneliness and quality of life. These findings suggest that eHealth literacy may function as a double-edged sword in later life. Future research is needed to clarify the underlying mechanisms and to examine how digital health competencies interact with psychosocial vulnerability in shaping older adults’ well-being.

## Introduction

Quality of life refers to an overall appraisal, both subjective and objective, based on an individual’s physical health, psychological state, social relationships, and living environment [1]. It encompasses both objective resources, such as health and economic conditions, and subjective experiences, including life satisfaction and well-being [2]. With accelerating population aging, the growing burden of chronic diseases and cognitive decline constrains social participation among older adults and increases their risk of reduced quality of life [3,4]. In China, rapid urbanization and changes in family structure are reshaping intergenerational relationships [5], with adult children increasingly living apart from their parents [6]. At the same time, family care and emotional support have weakened, and the proportion of older adults living alone has risen, together compounding the risk of poorer quality of life in later life [7]. Therefore, identifying the psychological and social determinants of quality of life in older adults is essential for designing targeted interventions and shaping effective pathways to healthy aging. In addition to quality of life, depressive symptoms represent a closely related psychological outcome that reflects emotional well-being and mental health status among older adults [8]. Previous studies have shown that loneliness is strongly associated with depressive symptoms and that both constructs capture important but complementary aspects of psychological vulnerability in later life [9,10]. Given this close conceptual linkage, depressive symptoms can serve as an alternative outcome to examine whether the observed associations are robust across related mental health indicators.

Loneliness is a subjective social emotional state that arises when individuals perceive a gap between the quantity or quality of their actual social relationships and their desired level of connection, leading to feelings of distress and loss [11]. Loneliness is now widely regarded as an important public health concern. A recent global systematic review estimated that the pooled prevalence of loneliness among community-dwelling older adults is about 32%, with substantial variation across regions and settings [12]. In China, survey data indicate that 36.6% of adults aged 60 years or older report some degree of loneliness, with higher levels observed among women, those living alone, individuals in poorer health, and those with lower socioeconomic status [13]. Previous studies have shown that loneliness is an important risk factor for a range of adverse health outcomes, including coronary heart disease and stroke [14], and is associated with a 26% to 50% increase in mortality risk [15,16]. The excess mortality attributable to loneliness is comparable in magnitude to that associated with physical inactivity or smoking [15]. Moreover, loneliness reduces subjective well-being and life satisfaction and further undermines quality of life in later life [17]. These findings highlight the need to treat loneliness as a key psychosocial determinant when examining quality of life in older adults and to clarify its pathways of influence to inform the design of targeted interventions.

eHealth literacy is commonly defined as the combined ability to locate, understand, appraise, and apply health information in internet-based and digital environments in support of health-related decision-making [18,19]. From the theoretical perspective, eHealth literacy may exert a bidirectional moderating influence on the relationship between loneliness and quality of life. According to the stress-buffering model, access to informational and emotional resources can mitigate the adverse impact of psychosocial stressors on health outcomes [20]. Within this framework, higher eHealth literacy could enable older adults experiencing loneliness to obtain trustworthy health information, access online social and community resources, and engage in self-management, thereby attenuating the detrimental effect of loneliness on quality of life. In contrast, frequent and intensive searching for health information online has been linked to heightened health anxiety and cyberchondria. Exposure to large volumes of risk-focused or conflicting information may generate additional stress and confusion [21]. For older adults who are already lonely and emotionally vulnerable, such patterns of information use may be associated with increased concerns about illness and a stronger negative association between loneliness and quality of life.

Therefore, the aims of this study are to (1) investigate the relationship between loneliness, eHealth literacy, and quality of life and (2) further explore the potential moderating role of eHealth literacy between loneliness and quality of life.

## Methods

### Participants and Procedure

This study was based on a community-based cross-sectional survey conducted among older adults aged 60 years and older in the Lanzhou New Area of Gansu Province, China. Lanzhou New Area is a national-level administrative district that oversees 7 community health service centers and 80 family physician teams. All 7 centers participated in the survey. Each family physician team was required to complete at least 20 household-based interviews. Among more than 30,000 older adults registered in the catchment areas of these centers, approximately 10% were randomly selected, yielding 2486 recruited participants in March 2025.

A standardized data collection procedure was implemented to maintain consistency across centers. Family physicians received centralized training before fieldwork. Questionnaires completed in less than 600 seconds were excluded, removing 290 responses. Another 86 individuals submitted duplicate entries and were therefore excluded. The final analytic sample consisted of 2110 participants..

### Measures

#### Loneliness

Loneliness was assessed using the Three-Item Loneliness Scale [22]. The scale consists of 3 items, each rated on a 3-point response format with “1= hardly ever,” “2= some of the time,” and “3= often.” Scores from the 3 items were summed to generate a total loneliness score ranging from 3 to 9. Higher scores indicate greater perceived loneliness. The internal consistency of the scale was high, with a Cronbach α of 0.872. The full-scale content is provided in Table S1 in Multimedia Appendix 1.

#### Quality of Life

Quality of life was measured using the brief Older People’s Quality of Life Questionnaire, which includes 13 statements covering core domains of well-being [23]. Participants indicated their level of agreement with each statement using a 5-point response scale ranging from strong disagreement to strong agreement. Item scores were summed to generate a total score ranging from 13 to 65, with higher values reflecting better quality of life. The scale demonstrated excellent internal consistency, with a Cronbach α of 0.975. The full list of items is available in Table S2 in Multimedia Appendix 1.

#### eHealth Literacy

The eHealth literacy was evaluated using an 8-item instrument [24]. Each item was rated on a 5-point Likert scale ranging from very inconsistent to very consistent. Scores were summed to produce a total ranging from 8 to 40, with higher values indicating stronger eHealth literacy. In this study, the eHealth literacy scale showed excellent internal reliability, with a Cronbach α of 0.980. Following prior studies, the total eHealth literacy score was treated as a continuous variable in the primary analyses [24,25]. In addition, the exploratory analyses were conducted to examine potential heterogeneity across groups of items within the scale. For this purpose, the 8 items of the eHealth literacy scale were grouped by the authors into 3 sets based on their content, reflecting applying literacy items (items 1‐5), critical literacy items (items 6‐7), and a decision-making literacy item (item 8). These item groupings were defined for exploratory purposes only and do not represent validated subscales of the instrument. Details of the instrument are presented in Table S3 in Multimedia Appendix 1.

#### Depressive Symptoms

Depressive symptoms were assessed using the 9-item Patient Health Questionnaire. This instrument contains 9 items that capture the frequency of core depressive experiences [26]. Each item is rated on a 4-level scale, ranging from 0 (not at all) to 3 (nearly every day), with response options of 1 (several days) and 2 (more than half of days). Scores were added to create a total ranging from 0 to 27, with higher values reflecting more pronounced depressive symptoms. The internal reliability of the scale was strong, with a Cronbach α of 0.921. Depressive symptoms were included as an alternative outcome variable in sensitivity analyses, replacing quality of life to assess the robustness of the primary interaction findings. Additional details are provided in Table S4 in Multimedia Appendix 1.

### Potential Confounders

Demographic and socioeconomic variables controlled for in the regression models included sex (male or female), age in years (60‐69 years, 70‐79 years, or ≥80 years), marital status (single, married, or partnered), education (less than high school or high school or above), employment status (not working or working), self-reported health status (bad, general, or good), chronic diseases (no or yes), smoking (no or yes), and alcohol consumption (no or yes).

### Statistical Analysis

Descriptive analyses were conducted to characterize the study sample. Continuous variables were presented as medians together with IQRs. Categorical variables were summarized using counts (n) and proportions (%).

Multiple linear regression models were fitted to examine the association between loneliness and quality of life. To evaluate the moderating role of eHealth literacy, interaction terms between loneliness and eHealth literacy were introduced. The primary analyses were conducted using the total eHealth literacy score as a continuous variable. Analyses based on item groupings of eHealth literacy were exploratory in nature and are presented in Multimedia Appendix 1. Prior to constructing the interaction terms, continuous variables involved in the interaction analyses were mean-centered to improve interpretability and reduce potential multicollinearity. To facilitate interpretation of the moderation effect, predicted values of quality of life were estimated at +1 or −1 SD of eHealth literacy, and the results were visualized. In addition, simple slope analyses were conducted to estimate the association between loneliness and quality of life at different levels of the continuous moderator. Model linearity assumptions were assessed by visually inspecting residual plots of continuous variables against regression residuals. The plots did not indicate substantial deviations from linearity. All analyses were performed using Stata (version 17.0; StataCorp). A 2-sided *P* value of <.05 was regarded as statistically significant.

To assess the robustness of the primary interaction findings based on the total eHealth literacy score, 2 sets of sensitivity analyses were performed. First, the outcome variable was replaced with depressive symptoms. Interaction models were reestimated with depressive symptoms as the outcome to examine the consistency of the moderating relationship. Second, stratified analyses were carried out with sex as the grouping variable. Robustness was evaluated based on whether the direction or statistical significance of the associations remained consistent after conducting stratified analyses [27].

### Ethical Considerations

Formal ethics approval was granted by the institutional review board of Peking University Third Hospital (20250224-01025-0129). Electronic informed consent was obtained from all study participants and assisting family physicians before participation. All data were collected and stored in a deidentified and anonymized form to protect participants’ privacy and confidentiality. Access to the data was restricted to authorized members of the research team only. Participation in this study was voluntary, and participants were informed of their right to withdraw at any time without consequences. No financial compensation was provided to participants.

## Results

### Descriptive Statistical Analysis

**Figure 1.**
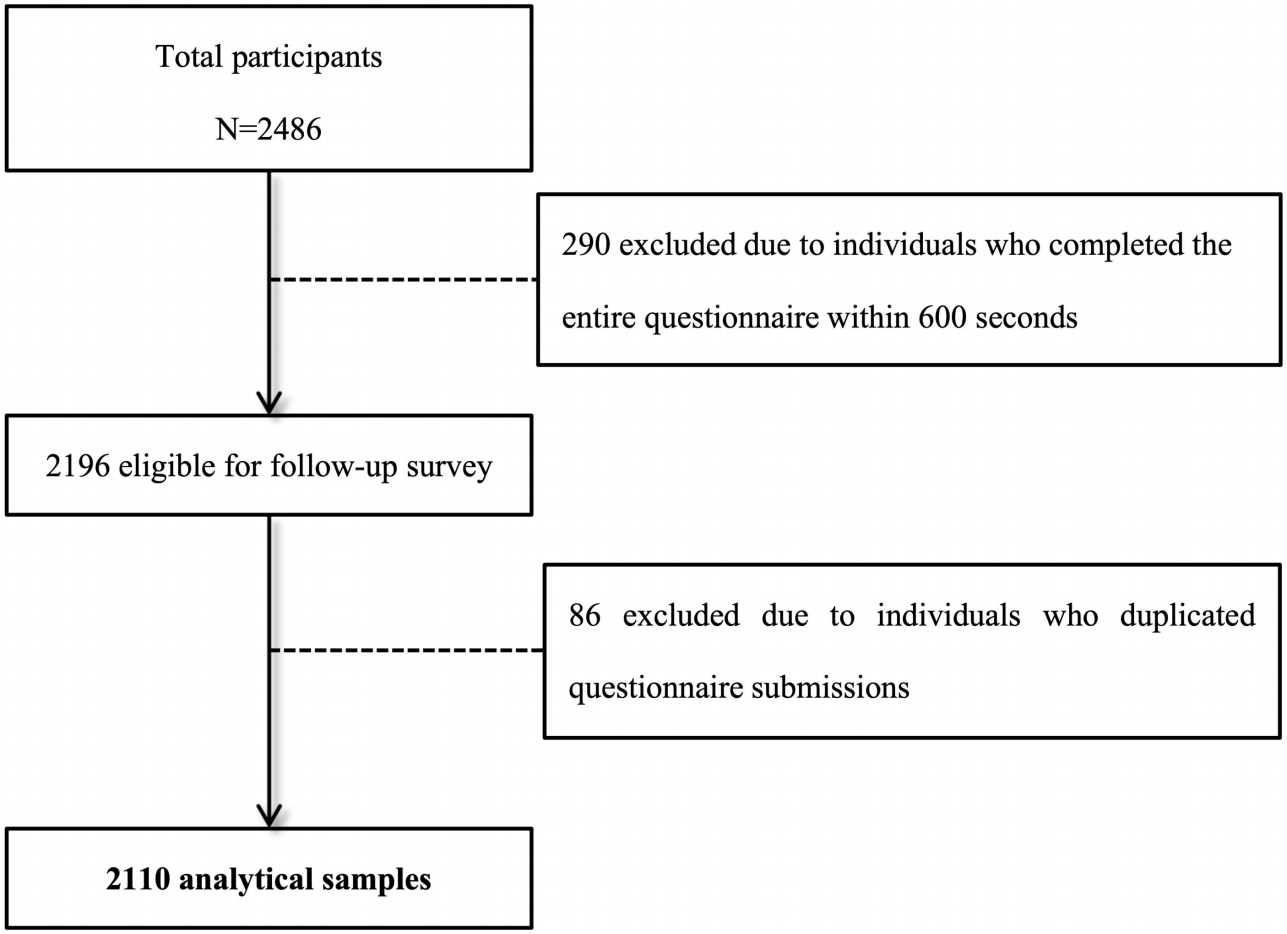
Flow diagram of participant screening and selection.

Figure 1 presents a flowchart of participant inclusion. Table 1 presents the baseline characteristics of the 2110 participants. Overall, loneliness, quality of life, eHealth literacy, and depressive symptoms were distributed at low to moderate levels. Of the 2110 participants, 1033 (48.9%) were female and 1077 (51.1%) were male. Most participants were aged 60 to 69 years (n=1132, 53.7%), followed by those aged 70 to 79 years (n=798, 37.8%). A large proportion were married or partnered (n=1778, 84.3%) and had education lower than high school (n=1802, 85.4%). The majority were not working (n=1919, 90.9%). Self-reported health was most commonly rated as general (n=1247, 59.1%). Chronic diseases were reported by 1232 (58.4%) participants. Most participants did not smoke (n=1536, 72.8%) and did not consume alcohol (n=1677, 79.5%).

**Table 1.** Descriptive statistics (N=2110).

Variables		Values
Loneliness, median (IQR); range		3.91 (3-5); 3‐9
Quality of life, median (IQR); range		46.35 (42-52); 13‐65
eHealth literacy, median (IQR); range		19.22 (8-26); 8‐40
Depressive symptoms, median (IQR); range		3.68 (0‐6); 0‐26
Sex, n (%)		
	Female	1033 (48.96)
	Male	1077 (51.04)
Age (y), n (%)		
	60‐69	1132 (53.65)
	70‐79	798 (37.82)
	≥80	180 (8.53)
Marital status, n (%)		
	Single	332 (15.73)
	Married or partnered	1778 (84.27)
Education, n (%)		
	Less than high school	1802 (85.4)
	High school or above	308 (14.6)
Employment status, n (%)		
	Not working	1919 (90.95)
	Working	191 (9.05)
Self-reported health status, n (%)		
	Bad	363 (17.2)
	General	1247 (59.1)
	Good	500 (23.7)
Chronic diseases, n (%)		
	No	878 (41.61)
	Yes	1232 (58.3)
Smoking, n (%)		
	No	1536 (72.8)
	Yes	574 (27.2)
Alcohol consumption, n (%)		
	No	1677 (79.48)
	Yes	433 (20.52)

### Relationship Between Loneliness, eHealth Literacy, and Quality of Life

Table 2 summarizes the results of the multiple linear regression models. In model 1, loneliness was significantly associated with lower quality of life (β=−0.66, 95% CI −1.03 to −0.29; *P*<.001). After adding eHealth literacy in model 2, this association remained significant and became slightly stronger (β=−0.83, 95% CI −1.18 to −0.49; *P*<.001). eHealth literacy was positively associated with quality of life (β=0.41, 95% CI 0.35-0.47; *P*<.001).

**Table 2.** Multiple linear regression analysis of the relationship between loneliness, eHealth literacy, and quality of life (N=2110).

Variables	Model 1: quality of life, β (robust SE^a^; 95% CI)		Model 2: quality of life, β (robust SE^b^; 95% CI)	
Loneliness	−0.66 (0.19; −1.03 to −0.29)^c^		−0.83 (0.18; −1.18 to −0.49)^c^	
eHealth literacy**^d^**	—^e^		0.41 (0.03; 0.35 to 0.47)^c^	
Sex (reference: female)				
Male	−0.33 (0.69; −1.67 to 1.02)		−0.43 (0.65; −1.70 to 0.85)	
Age (years; ref: 60-69)				
70-79	−1.62 (0.58; −2.76 to −0.47)^f^		−0.64 (0.56; −1.75 to 0.47)	
≥80	−1.03 (0.95; −2.89 to 0.84)		0.71 (0.96; −1.17 to 2.59)	
Marital status (ref: single)				
Married or partnered	2.04 (0.79; 0.49 to 3.59)^g^		1.10 (0.77; −0.41 to 2.61)	
Education (ref: less than high school)				
High school or above	0.50 (0.76; −0.98 to 1.98)		−1.09 (0.70; −2.46 to 0.28)	
Self-reported health status (ref: bad)				
General	3.13 (0.72; 1.71 to 4.55)^c^		2.80 (0.70; 1.44 to 4.17)^c^	
Good	7.07 (0.87; 5.36 to 8.78)^c^		6.30 (0.84; 4.65 to 7.95)^c^	
Employment status (ref: not working)				
Working	−0.39 (0.96; −2.27 to 1.49)		−1.02 (0.86; −2.71 to 0.67)	
Chronic diseases (ref: no)				
Yes	−0.55 (0.54; −0.50 to 1.61)		0.55 (0.51; −0.46 to 1.55)	
Smoking (ref: no)				
Yes	0.11 (0.75; −1.36 to 1.58)		0.29 (0.72; −1.12 to 1.70)	
Alcohol consumption (ref: no)				
Yes	0.98 (0.75; −0.53 to 2.46)		0.46 (0.71; −0.94 to 1.86)	
Constant	43.97 (1.50; 41.03 to 46.90)^c^		45.69 (1.41; 42.93 to 48.45)^c^	

a *R*2=0.0658.

b *R*2=0.1521.

c *P*<.001.

d The continuous variable of eHealth literacy has been mean-centered.

e Variables were not included in the model.

f *P*<.01.

g *P*<.05.

Exploratory analyses based on author-defined item groupings of eHealth literacy were conducted to examine their associations with quality of life. The results showed broadly consistent patterns with the primary analyses based on the total eHealth literacy score. Detailed results are presented in Table S5 in Multimedia Appendix 1.

### Results of Multiple Linear Regression With Interaction Terms

Table 3 shows the results of the interaction models. In model 6, the interaction between loneliness and eHealth literacy was significant (β=−0.04, 95% CI −0.08 to −0.002; *P*=.04), indicating that higher eHealth literacy was associated with a steeper negative association between loneliness and quality of life. The inclusion of eHealth literacy and its interaction with loneliness increased the explained variance in quality of life from 6.58% (model 1, *R*²=0.0658) to 15.37% (model 6, *R*²=0.1537), indicating improved model explanatory power (Δ*R*²=0.0879). When comparing the model with main effects to the interaction model, the incremental contribution of the interaction term was 0.0016 (model 6, *R*²=0.1537; model 2, *R*²=0.1521; Δ*R*²=0.0016), and the interaction effect was statistically significant. Additional exploratory analyses examined interaction effects between loneliness and the author-defined item groupings of eHealth literacy. The patterns were consistent with the primary interaction findings based on the total score. Detailed results are presented in Table S6 in Multimedia Appendix 1.

**Table 3.** Results of multiple linear regression with interaction terms examining the relationship between loneliness, eHealth literacy, and quality of life (N=2110).

Variables	Model 6: quality of life, β (robust SE; 95% CI)^a^	
Loneliness	−0.83 (0.18; −1.18 to −0.49)^b^	
eHealth literacy**^c^**	0.56 (0.08; 0.40 to 0.73)^b^	
Interaction		
Loneliness×eHealth literacy**^c^**	−0.04 (0.02; −0.08 to −0.002)^d^	
Covariates^e^		
Constant	45.64 (1.42; 42.86 to 48.41)^b^	

a *R*2=0.1537.

b *P*<.001.

c The continuous variable of eHealth literacy has been mean-centered.

d *P*<.05.

e Adjusted for all covariates.

**Figure 2.**
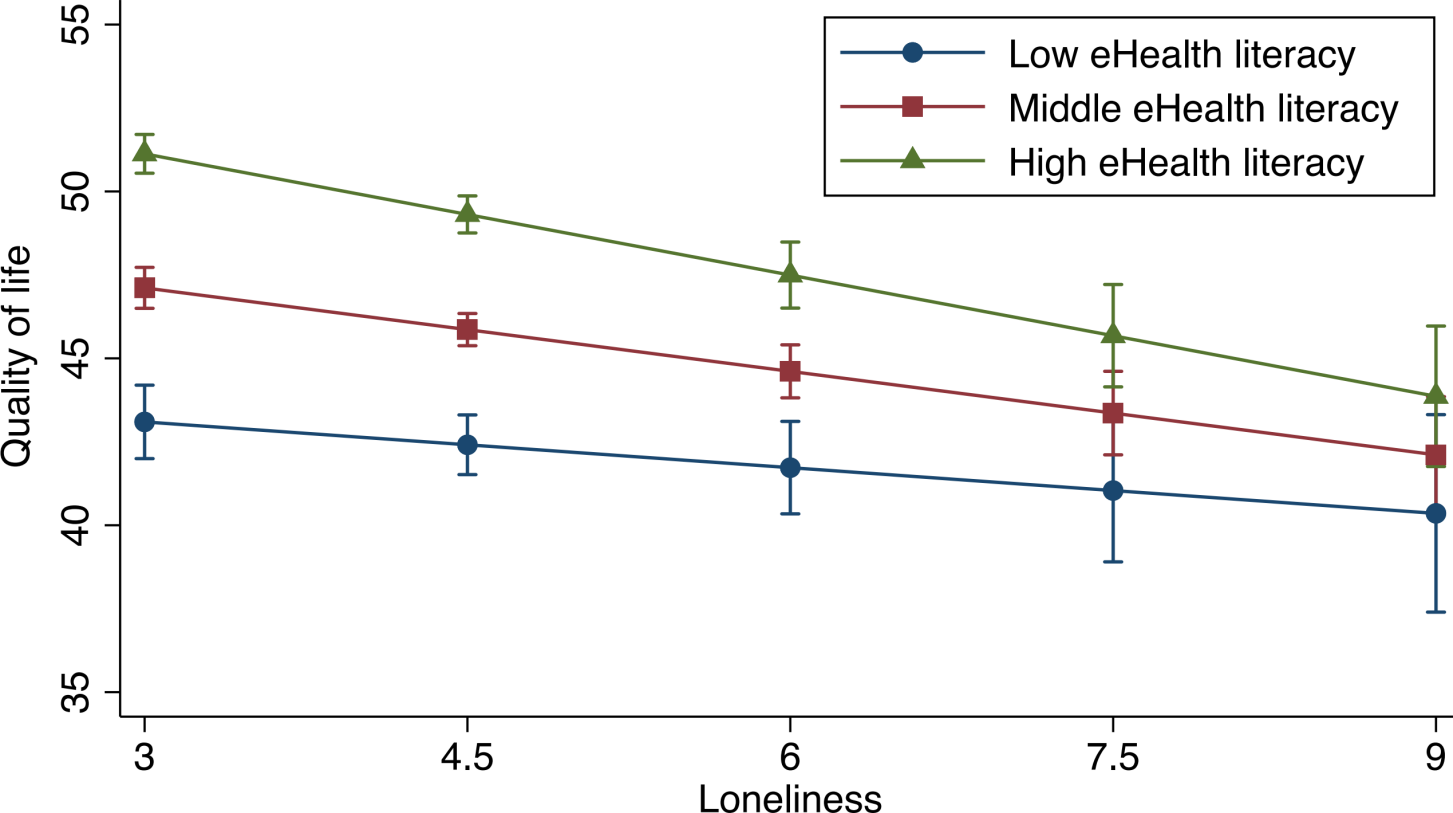
Moderating effect of eHealth literacy on the relationship between loneliness and quality of life.

To further interpret the interaction between loneliness and eHealth literacy, predicted values of quality of life were estimated at different levels of the continuous moderator. Figure 2 presents the interaction plot based on mean-centered eHealth literacy, with predicted values shown at low (mean−1 SD), middle, and high (mean+1 SD) levels of eHealth literacy. The results indicate that quality of life declined as loneliness increased across all levels of eHealth literacy. Simple slope analyses showed that the association between loneliness and quality of life was not statistically significant at low levels of eHealth literacy (simple slope=−0.46; *P*=.13), but became significantly stronger at the middle levels (simple slope=−0.83; *P*<.001) and high levels of eHealth literacy (simple slope=−1.21; *P*<.001). These findings suggest that the negative association between loneliness and quality of life becomes more pronounced as eHealth literacy increases.

### Sensitivity Analysis

Table S7 in Multimedia Appendix 1 shows the results of the interaction models when depressive symptoms were used as the outcome. Loneliness remained strongly associated with higher depressive symptoms across all models. The interaction between loneliness and eHealth literacy was significant and positive, suggesting that higher eHealth literacy was associated with a steeper positive association between loneliness and depressive symptoms (model 10). Figure S1 in Multimedia Appendix 1 illustrates the simple slopes of loneliness on depressive symptoms at different levels of the continuous eHealth literacy moderator. Depressive symptoms increased with higher levels of loneliness across all levels of eHealth literacy (low eHealth literacy, simple slope=1.62; *P*<.001 vs middle eHealth literacy, simple slope=1.84; *P*<.001 vs high eHealth literacy, simple slope=2.06; *P*<.001). The slope was steeper at higher levels of eHealth literacy, indicating that the positive association between loneliness and depressive symptoms became stronger as eHealth literacy increased. These results indicate that the moderating role of eHealth literacy remained stable when the outcome variable was replaced, supporting the robustness of the study findings.

Table S8 in Multimedia Appendix 1 presents the stratified results. In both male and female participants, eHealth literacy continued to show a positive association with quality of life. The interaction between loneliness and eHealth literacy was significant among female participants (β=−0.07, 95% CI −0.13 to −0.01; *P*=.02). Overall, the stratified models showed that the interaction results were consistent with the primary interaction findings based on the total eHealth literacy score, indicating the robustness of the findings.

## Discussion

### Principal Findings

This study examined loneliness, eHealth literacy, and quality of life among older adults in China and assessed the moderating role of eHealth literacy. Loneliness showed a clear negative association with quality of life. Higher eHealth literacy was positively associated with quality of life. Interaction analyses indicated that higher eHealth literacy was associated with a steeper negative association between loneliness and quality of life among older adults.

This study found a consistent negative association between loneliness and quality of life among older adults. This result aligns with evidence from cohort studies and systematic reviews showing that loneliness is linked to depression, anxiety, physical decline, cardiovascular events, and higher all-cause mortality, as well as sustained reductions in subjective well-being and health-related quality of life [10,15,17,28]. Persistent loneliness may impair psychological and social functioning through pathways involving functional deterioration [29], negative emotional accumulation, and reduced social participation, thereby undermining quality of life. These findings highlight the importance of prioritizing loneliness reduction in healthy aging policies and community interventions aimed at improving quality of life in later life.

We observed a robust positive association between eHealth literacy and quality of life in older adults. Existing evidence indicates that higher eHealth literacy enhances the ability of older adults to obtain and understand online health information [30], supports the use of digital health services, and promotes self-management [31], which can improve both psychological and physical health and ultimately enhance quality of life [32]. In China, where health care resources are unevenly distributed and accessing offline services can be burdensome, older adults with higher eHealth literacy are more likely to secure timely information and support through digital channels, translating digital access advantages into better quality of life [30,33].

Our study also showed that higher eHealth literacy was associated with a steeper negative association between loneliness and quality of life. This pattern differs from prior evidence, particularly from studies conducted during the COVID-19 pandemic among younger or student populations, where higher eHealth literacy was more commonly found to attenuate the negative associations between fear, loneliness, depressive symptoms, and health-related quality of life [34-37]. These findings suggest that eHealth literacy may have dual effects in digital health environments. Higher eHealth literacy may be accompanied by more intensive health information seeking and greater reliance on self-directed health management, which can increase cognitive load and decision burden [18,31]. Under conditions of loneliness and limited emotional support, greater engagement with online health information may translate into increased tendencies toward cyberchondria and psychological strain, thereby strengthening the association between loneliness and reduced quality of life [38,39]. In addition, in the absence of adequate family support or professional guidance, older adults with high eHealth literacy may also need to interpret complex health information on their own, which can intensify confusion, stress, and uncertainty and create a cumulative negative effect between loneliness and digital information exposure [40]. Furthermore, the cross-sectional design precludes conclusions regarding temporal ordering. It is possible that older adults with poorer well-being or heightened health concerns are more motivated to engage with online health information and to develop or report higher eHealth literacy, while loneliness simultaneously contributes to lower quality of life. This possibility highlights the need for longitudinal and experimental research to clarify directionality and underlying mechanisms. These findings highlight the need to further investigate the context-dependent functions of eHealth literacy and to examine potential mediating mechanisms, including information overload, health anxiety, cyberchondria, and decision burden, through longitudinal and experimental designs that can clarify temporal ordering and causal pathways. Additionally, strategies aimed at enhancing digital competence among older adults may require complementary forms of structured support, such as assistance in interpreting complex online health information and facilitated linkage to trusted community or primary care resources, particularly for individuals experiencing social vulnerability.

#### Strengths

This study has several strengths. It draws on a large community-based sample of older adults from multiple health service centers, providing robust evidence from a population that is often underrepresented in digital health research. The study simultaneously examined loneliness, eHealth literacy, and quality of life, allowing for a nuanced understanding of how digital competencies relate to quality of life in later life. The use of validated instruments and rigorous quality control procedures strengthened the reliability of the measures. In addition, the inclusion of interaction models and sensitivity analyses enhanced the robustness of the findings. By identifying a pattern in which higher eHealth literacy was associated with a steeper negative association between loneliness and quality of life, this study provides new insights into the complex role of eHealth literacy and contributes to the understanding of digital vulnerability among older adults.

#### Limitations

This study has several limitations. First, the cross-sectional design precludes establishing temporal ordering. Future studies would benefit from longitudinal designs to clarify how loneliness and eHealth literacy interact over time and to identify potential bidirectional pathways. Second, all variables were measured through self-report, which may introduce reporting bias. Future research could incorporate clinical indicators, digital trace data, or performance-based assessments to provide more objective measures of health status and online information use. Third, the sample was drawn from one region in northwestern China, and findings may not generalize to areas with different digital access or social support structures, underscoring the value of multisite or nationally representative studies.

### Conclusions

This study found that loneliness was consistently associated with lower quality of life among older adults and that higher eHealth literacy was associated with a steeper negative association between loneliness and quality of life. These results suggest that eHealth literacy functions as a dual-edged resource in later life. Although higher literacy is linked to better quality of life overall, it may intensify the challenges faced by lonely older adults when navigating complex or risk-oriented online health information. Given the cross-sectional and observational nature of the study, these interpretations should be considered exploratory. Future research is needed to examine the underlying psychological mechanisms, including potential roles of health anxiety, information overload, or emotional regulation processes, and to determine whether complementary support strategies could mitigate unintended negative consequences in digitally engaged but socially vulnerable populations.

## Supplementary material

10.2196/88600 Multimedia Appendix 1 Scales used in study and additional statistical results.
