# Supplementary material for: Association Between Loneliness, eHealth Literacy, and Quality of Life Among Chinese Older Adults: Cross-Sectional Study
Source: JMIR Aging. 2026 May 11;9:e88600. doi: 10.2196/88600 (PMC13346640; doi:10.2196/88600)
Supplement: Multimedia Appendix 1 [file aging-v9-e88600-s001.docx]

**Supplementary materials**

**Table S1.** Three item loneliness scale.

| **Item** | **Response Options** |
| --- | --- |
| How often do you feel that you lack companionship? | 1 = Hardly ever  2 = Some of the time  3 = Often |
| How often do you feel left out? |  |
| How often do you feel isolated from others? |  |

**Table S2.** Brief Older People’s Quality of Life questionnaire (OPQOL-brief).

| **Item** | **Response Options** |
| --- | --- |
| I enjoy my life overall. | 1 = Strongly disagree  2 = Disagree  3 = Undecided  4 = Agree  5 = Strongly agree |
| I look forward to things. |  |
| I am healthy enough to get out and about. |  |
| My family, friends or neighbors would help me if needed. |  |
| I have social or leisure activities/hobbies that I enjoy doing. |  |
| I try to stay involved with things. |  |
| I am healthy enough to have my independence. |  |
| I can please myself what I do. |  |
| I feel safe where I live. |  |
| I get pleasure from my home. |  |
| I take life as it comes and make the best of things. |  |
| I feel lucky compared to most people. |  |
| I have enough money to pay for household bills. |  |

**Table S3.** eHealth literacy scale.

| **Item-group** | **Item** | **Response Options** |
| --- | --- | --- |
| Applying literacy | I know what health resources are available on the Internet. | 1 = Strongly disagree  2 = Disagree  3 = Undecided  4 = Agree  5 = Strongly agree |
|  | I know where to find helpful health resources on the Internet. |  |
|  | I know how to find helpful health resources on the Internet. |  |
|  | I know how to use the Internet to answer my questions about health. |  |
|  | I know how to use the health information I find on the Internet to help me. |  |
| Critical literacy | I have the skills I need to evaluate the health resources I find on the Internet. |  |
|  | I can tell high quality health resources from low quality health resources on the Internet. |  |
| Decision-making literacy | I feel confident in using information from the Internet to make health decisions. |  |

**Table S4.** Patient health questionnaire-9 (PHQ-9).

| **Item** | **Response Options** |
| --- | --- |
| Little interest or pleasure in doing things. | 0 = Not at all  1 = Several days  2 = More than half the days  3 = Nearly every day |
| Feeling down, depressed, or hopeless. |  |
| Trouble falling or staying asleep, or sleeping too much. |  |
| Feeling tired or having little energy. |  |
| Poor appetite or overeating. |  |
| Feeling bad about yourself-or that you are a failure or have let yourself or your family down. |  |
| Trouble concentrating on things, such as reading or watching television. |  |
| Moving or speaking slowly, or being fidgety/restless. |  |
| Thoughts that you would be better off dead, or of hurting yourself. |  |

**Table S5.** Multiple linear regression on the relationship between loneliness, item groupings of eHealth literacy and quality of life (N=2,110).

| **Variables** | **Model 3**  **Quality of Life** | | **Model 4**  **Quality of Life** | | **Model 5**  **Quality of Life** | |
| --- | --- | --- | --- | --- | --- | --- |
|  | **β (Robust S.E.)** | **95% CI** | **β (Robust S.E.)** | **95% CI** | **β (Robust S.E.)** | **95% CI** |
| **Loneliness** | -0.83 (0.18) *** | [-1.17, -0.49] | -0.82 (0.18) *** | [-1.16, -0.47] | -0.81 (0.18) *** | [-1.16, -0.46] |
| **Applying literacy ^a^** | 0.64 (0.05) *** | [0.54, 0.73] | -- | -- | -- | -- |
| **Critical literacy ^a^** | -- | -- | 1.48 (0.11) *** | [1.26, 1.70] | -- | -- |
| **Decision-making literacy ^a^** | -- | -- | -- | -- | 2.89 (0.22) *** | [2.45, 3.33] |
| **Covariates** | Control |  | Control |  | Control |  |
| **Con.** | 45.68 (1.41) *** | [42.91, 48.45] | 45.51 (1.42) *** | [42.73, 48.29] | 45.34 (1.41) *** | [42.57, 48.11] |
| **R^2^** | 0.1506 |  | 0.1418 |  | 0.1420 |  |

Note. a, the continuous variables of Applying literacy, Critical literacy and Decision-making literacy have been mean-centered; β, coefficient; S.E., standard error; CI, confidence interval, *P<0.05 **P<0.01 ***P<0.001.

**Table S6.** Results of multiple linear regression with interaction on the relationship between loneliness, item groupings of eHealth literacy and quality of life (N=2,110).

| **Variables** | **Model 7**  **Quality of Life** | | **Model 8**  **Quality of Life** | | **Model 9**  **Quality of Life** | |
| --- | --- | --- | --- | --- | --- | --- |
|  | **β (Robust S.E.)** | **95% CI** | **β (Robust S.E.)** | **95% CI** | **β (Robust S.E.)** | **95% CI** |
| **Loneliness** | -0.83 (0.18) *** | [-1.18, -0.48] | -0.82 (0.18) *** | [-1.17, -0.47] | -0.81 (0.18) *** | [-1.16, -0.46] |
| Applying literacy ^a^ | 0.87 (0.13) *** | [0.62, 1.13] | -- | -- | -- | -- |
| Critical literacy ^a^ | -- | -- | 2.10 (0.32) *** | [1.47, 2.74] | -- | -- |
| Decision-making literacy ^a^ | -- | -- | -- | -- | 4.11 (0.65) *** | [2.84, 5.38] |
| **Interaction** |  |  |  |  |  |  |
| Loneliness × Applying literacy | -0.06 (0.03) * | [-0.13, -0.002] | -- | -- | -- | -- |
| Loneliness × Critical literacy | -- | -- | -0.17 (0.08) * | [-0.32, -0.01] | -- | -- |
| Loneliness × Decision-making literacy | -- | -- | -- | -- | -0.32 (0.15) * | [-0.62, -0.03] |
| **Covariates** | Control |  | Control |  | Control |  |
| **Con.** | 45.63 (1.42) *** | [42.85, 48.41] | 45.46 (1.43) *** | [42.67, 48.26] | 45.29 (1.42) *** | [42.50, 48.08] |
| **R^2^** | 0.1521 |  | 0.1435 |  | 0.1438 |  |

Note. a, the continuous variables of Applying literacy, Critical literacy and Decision-making literacy have been mean-centered; β, coefficient; S.E., standard error; CI, confidence interval, *P<0.05 **P<0.01 ***P<0.001.

**Table S7.** Results of multiple linear regression with interaction on the relationship between loneliness, eHealth literacy, item groupings of eHealth literacy and depressive symptoms (N=2,110).

| **Variables** | **Model 10**  **Depressive Symptoms** | | **Model 11**  **Depressive Symptoms** | | **Model 12**  **Depressive Symptoms** | | **Model 13**  **Depressive Symptoms** | |
| --- | --- | --- | --- | --- | --- | --- | --- | --- |
|  | **β (Robust S.E.)** | **95% CI** | **β (Robust S.E.)** | **95% CI** | **β (Robust S.E.)** | **95% CI** | **β (Robust S.E.)** | **95% CI** |
| **Loneliness** | 1.84 (0.07) *** | [1.69, 1.98] | 1.84 (0.07) *** | [1.70, 1.98] | 1.84 (0.07) *** | [1.70, 1.98] | 1.84 (0.07) *** | [1.69, 1.98] |
| **eHealth literacy ^a^** | -0.07 (0.03) ** | [-0.13, -0.02] | -- | -- | -- | -- | -- | -- |
| Applying literacy | -- | -- | -0.11 (0.04) * | [-0.20, -0.02] | -- | -- | -- | -- |
| Critical literacy | -- | -- | -- | -- | -0.33 (0.11) ** | [-0.53, -0.12] | -- | -- |
| Decision-making literacy | -- | -- | -- | -- | -- | -- | -0.52 (0.21) * | [-0.93, -0.11] |
| **Interaction** |  |  |  |  |  |  |  |  |
| **Loneliness × eHealth literacy ^a^** | 0.02 (0.01) ** | [0.01, 0.04] |  |  |  |  |  |  |
| Loneliness × Applying literacy | -- | -- | 0.04 (0.01) ** | [0.01, 0.06] | -- | -- | -- | -- |
| Loneliness × Critical literacy | -- | -- | -- | -- | 0.10 (0.03) ** | [0.04, 0.16] | -- | -- |
| Loneliness × Decision-making literacy | -- | -- | -- | -- | -- | -- | 0.16 (0.06) ** | [0.04, 0.28] |
| **Covariates** | Control |  | Control |  | Control |  | Control |  |
| **Con.** | -2.28 (0.47) *** | [-3.19, -1.36] | -2.28 (0.47) *** | [-3.19, -1.37] | -2.27 (0.47) *** | [-3.19, -1.36] | -2.31 (0.47) *** | [-3.22, -1.39] |
| **R^2^** | 0.4124 |  | 0.4208 |  | 0.4227 |  | 0.4200 |  |

Note. a, the continuous variable of eHealth literacy (with item groupings of eHealth literacy) has been mean-centered; β, coefficient; S.E., standard error; CI, confidence interval, *P<0.05 **P<0.01 ***P<0.001.

**Table S8.** Interaction models stratification by sex.

| **Variables** | **Model 14 (Male)**  **Quality of Life** | | **Model 15 (Female)**  **Quality of Life** | |
| --- | --- | --- | --- | --- |
|  | **β (Robust S.E.)** | **95% CI** | **β (Robust S.E.)** | **95% CI** |
| **Loneliness** | -1.25 (0.28) | [-1.80, -0.69] | -0.54 (0.23) * | [-0.44, 2.04] |
| **eHealth literacy ^a^** | 0.45 (0.12) *** | [0.22, 0.69] | 0.66 (0.13) *** | [0.41, 0.90] |
| **Interaction** |  |  |  |  |
| Loneliness × eHealth literacy **^a^** | -0.005 (0.03) | [-0.06, 0.05] | -0.07 (0.03) * | [-0.13, -0.01] |
| **Covariates** | Control |  | Control |  |
| **Con.** | 47.30 (2.54) *** | [42.31, 52.29] | 43.96 (1.77) *** | [40.48, 47.43] |
| **R^2^** | 0.1647 |  | 0.1546 |  |

Note. a, the continuous variable of eHealth literacy has been mean-centered; β, coefficient; S.E., standard error; CI, confidence interval, *P<0.05 **P<0.01 ***P<0.001.


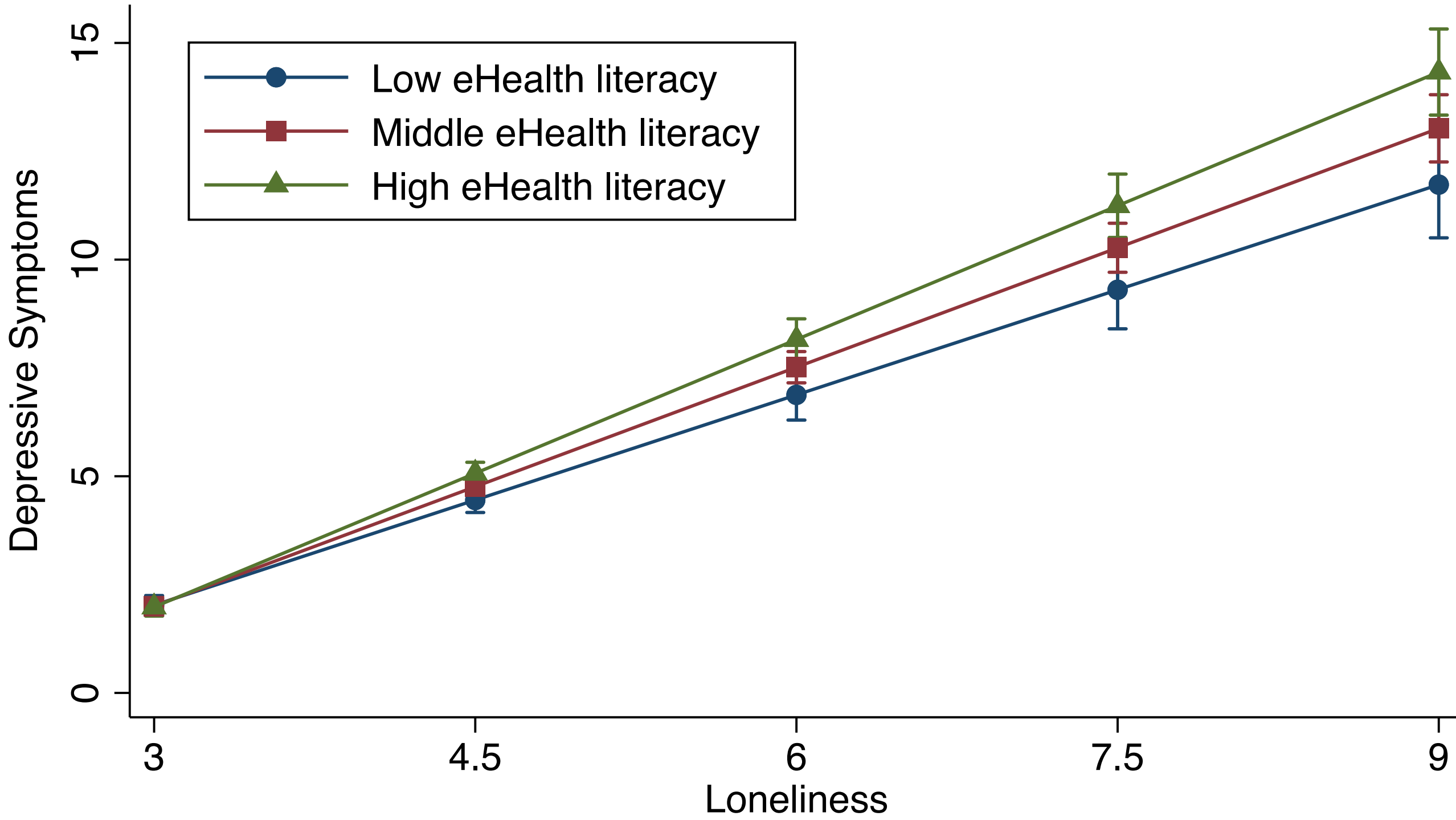


**Figure S1.** Moderation of eHealth literacy on the relationship between loneliness and depressive symptoms.
